# Self-reported tobacco smoking practices among medical students and their perceptions towards training about tobacco smoking in medical curricula: A cross-sectional, questionnaire survey in Malaysia, India, Pakistan, Nepal, and Bangladesh

**DOI:** 10.1186/1747-597X-5-29

**Published:** 2010-11-16

**Authors:** Chandrashekhar T Sreeramareddy, Sushil Suri, Ritesh G Menezes, HN Harsha Kumar, Mahbubur Rahman, Md R Islam, Xavier V Pereira, Mohsin Shah, Brijesh Sathian, Ullasa Shetty, Vina R Vaswani

**Affiliations:** 1 Department of Community Medicine, Melaka Manipal Medical College, Melaka, Malaysia (Manipal University; 2 Department of Internal Medicine, Melaka Manipal Medical College, Malaysia (Manipal University; 3 Department of Forensic Medicine, Kasturba Medical College, Melaka, Mangalore, India (Manipal University; 4 Department of Community Medicine, Kasturba Medical College, Mangalore, India (Manipal University; 5 Department of Community Medicine, Faridpur Medical College, Faridpur, Bangladesh; 6 CMH Lahore Medical College, University of Health Sciences, Lahore, Pakistan; 7 Department of Community Medicine, Manipal College of Medical Sciences, Pokhara, Nepal; 8 Department of Psychiatry, Melaka Manipal Medical College, Melaka, Malaysia (Manipal University; 9 Department of Forensic Medicine and Toxicology, AJ Institute of Medical Sciences, Mangalore, India; 10 Department of Forensic Medicine and Toxicology, Yenepoya Medical College, Mangalore, India Affiliated to Manipal University

## Abstract

**Background:**

Tobacco smoking issues in developing countries are usually taught non-systematically as and when the topic arose. The World Health Organisation and Global Health Professional Student Survey (GHPSS) have suggested introducing a separate integrated tobacco module into medical school curricula. Our aim was to assess medical students' tobacco smoking habits, their practices towards patients' smoking habits and attitude towards teaching about smoking in medical schools.

**Methods:**

A cross-sectional questionnaire survey was carried out among final year undergraduate medical students in Malaysia, India, Nepal, Pakistan, and Bangladesh. An anonymous, self-administered questionnaire included items on demographic information, students' current practices about patients' tobacco smoking habits, their perception towards tobacco education in medical schools on a five point Likert scale. Questions about tobacco smoking habits were adapted from GHPSS questionnaire. An *'ever smoker' *was defined as one who had smoked during lifetime, even if had tried a few puffs once or twice. 'Current smoker' was defined as those who had smoked tobacco product on one or more days in the preceding month of the survey. Descriptive statistics were calculated.

**Results:**

Overall response rate was 81.6% (922/1130). Median age was 22 years while 50.7% were males and 48.2% were females. The overall prevalence of 'ever smokers' and 'current smokers' was 31.7% and 13.1% respectively. A majority (> 80%) of students asked the patients about their smoking habits during clinical postings/clerkships. Only a third of them did counselling, and assessed the patients' willingness to quit. Majority of the students agreed about doctors' role in tobacco control as being role models, competence in smoking cessation methods, counseling, and the need for training about tobacco cessation in medical schools. About 50% agreed that current curriculum teaches about tobacco smoking but not systematically and should be included as a separate module. Majority of the students indicated that topics about health effects, nicotine addiction and its treatment, counselling, prevention of relapse were important or very important in training about tobacco smoking.

**Conclusion:**

Medical educators should consider revising medical curricula to improve training about tobacco smoking cessation in medical schools. Our results should be supported by surveys from other medical schools in developing countries of Asia.

## Background

Tobacco use is one of the leading preventable causes of premature death, disease and disability around the world [1]. Tobacco use is one of the risk factors for six out of eight leading causes of death worldwide [2]. An estimated 4.9 million deaths occurring annually can be attributed to tobacco use. This may increase to 10 million by the year 2020, if the current tobacco use epidemic continues and more than 70% of these deaths are expected to occur in developing countries [3]. Medical students who are future doctors have an important role to be played in tobacco cessation and prevention efforts. On the contrary, a vast body of evidence shows that prevalence of tobacco smoking is fairly high among medical students. Ironically medical students themselves lack adequate knowledge about smoking-related diseases and tobacco cessation techniques [4].

Expert reviews have suggested that undergraduate medical students should be equipped with knowledge and skills to promote smoking cessation skills among their future patients [4-7]. However, a worldwide medical school survey on teaching about tobacco has reported that tobacco smoking issues are usually taught non-systematically as and when the topic arose. The survey also reported that only a tenth of surveyed medical schools had a specific tobacco module. Another tenth of medical schools located mainly in Africa and Asia do not teach about tobacco issues [8]. Further, Global Health Professionals Student Survey (GHPSS) has suggested introducing a separate integrated tobacco module in medical schools to augment other strategies of tobacco control [9,10]. A few studies about medical students training in tobacco cessation and prevention methods have been reported from developed countries [11-13]. Such studies are seldom reported from South Asian countries [8]. A multi-country survey (1986-89) about habits, knowledge and attitudes of medical students regarding tobacco was carried out by the International Union against Tuberculosis and Lung Diseases (IUATLD) in Europe, Africa, Middle-East and Asia [14-16]. The survey has reported that medical students lack knowledge about smoking cessation and preventive measures. In a study from Bahrain, primary care physicians reported that training about smoking cessation techniques was not given in their medical schools [17].

Considering the pitfalls in training medical students about tobacco smoking in Asian countries and the recommendations made by GHPSS, we thought it was imperative to assess prevailing practices of medical students about tobacco smoking, prevention and cessation techniques, their perceptions and attitudes towards training in medical schools. Since country profiles, chronic disease burden and medical education systems are similar in the region of South Asia, we aimed to carry out an exploratory survey about training of medical students in tobacco-related issues in five South Asian countries. The objectives of our survey were as follows:

1. To assess the current practices of medical students towards tobacco smoking habits among their patients during clinical postings/clerkships.

2. To assess the perceptions and attitudes of medical students about their training in tobacco smoking.

3. To determine the current tobacco use among medical students.

## Methods

### Study design

A cross-sectional, self-administered anonymous questionnaire-based survey was carried out.

### Setting and Participants

Final year undergraduate medical students were selected for this survey. The students studying in final year were expected to have completed nearly two years or more of clinical rotational postings in various medical specialties. The participants were selected from the following medical schools in Malaysia, India, Pakistan, Bangladesh, and Nepal: Melaka-Manipal Medical College (MMMC), Malaysia (a medical twinning program affiliated to Manipal University, India), Yenepoya Medical College (YMC) affiliated to Yenepoya University and AJ Institute of Medical Sciences (AJIMS), Mangalore, India (affiliated to Rajiv Gandhi University of Health Sciences, Karnataka, India), Manipal College of Medical Sciences, Pokhara (MCOMS), Nepal (affiliated to Kathmandu University), Combined Military Hospital, Lahore Medical College (CMH, LMC), (affiliated to the University of Health Sciences, Lahore) Pakistan and Faridpur Medical College (FMC), Faridpur, Bangladesh (affiliated to the University of Dhaka).

The undergraduate medical course in the medical schools we surveyed is of four to five years duration with compulsory rotational internships after completion of the final qualifying examination. Medical curricula were mostly traditional, lecture-based except at MCOMS (Nepal), where problem-based learning (PBL) was emphasised and at MMMC (Malaysia) emphasis was laid on PBL and tutorials rather than lectures alone. In all the medical schools, clinical rotations start from the third year onwards. Students undergo clinical training in major medical, surgical and allied specialties during each academic year. Duration of clinical rotations is usually four to eight weeks. At MMMC students undergo clerkships in major medical and surgical specialties during fifth year.

### Sampling and sample size

Since our survey was exploratory in nature, a convenient sample of medical school/s was selected from in each country. All medical students studying in the final year of the undergraduate medical course were included for the survey.

### Questionnaire

After a detailed review of literature and informal discussions with students about the topic, we developed a structured questionnaire in English (Annexure-1). The questionnaire was pretested among 20 medical students in each of the medical schools where the survey was planned. During pretesting, students also gave a written feedback about the questionnaire. We modified the questionnaire based on pretest results and students' written feedback. In the final questionnaire, the first section contained instructions, and a statement about confidentiality of information to be provided. Subsequent sections were about demographic information, students' current practices about tobacco smoking habits among their patients seen during clinical postings, their attitudes (in a five point Likert scale) towards teaching about tobacco smoking in their curriculum. Medical students' practices about their patients' smoking habits were assessed as 'never' to 'always', their perceptions towards training about smoking in their medical school as 'strongly disagree' to 'strongly agree' and the rating of contents in the tobacco module as 'unimportant' to 'very important'. We also included some questions about medical students' tobacco smoking habits. These questions were adapted and modified from the Global Health Professionals Student Survey (GHPSS) core questionnaire [18]. An *'ever smoker' *was defined as one who had smoked during lifetime, even if had tried a few puffs, once or twice. A *'current smoker' *was defined as one who had smoked during 30 days prior to the survey including the ones who smoked every day [19].

### Data collection

Ethical approval and/or permission to carry out the survey were obtained from each medical school. Between November, 2009 and May, 2010 questionnaire was administered by the collaborators who were working as teaching faculty at each site. At each site, the students were briefed about the purpose of the research and were invited to participate in the survey. The students were informed that their participation in the survey was anonymous, voluntary and was not compulsory. Assurance was given about anonymity and confidentiality of the information to be provided. Informed consent was sought from all the students in the questionnaire. However, to maintain anonymity the students who participated in the survey signed a separate sheet containing their names and roll numbers. They were also instructed that they should not enter any identifiable personal information in the questionnaire. The questionnaire was distributed to the students during small group teaching sessions such as student seminars, tutorials, self-directed learning, problem-based learning etc. Completed questionnaires were collected.

### Data management and statistical analysis

We used SPSS (Statistical Package for Social Sciences) version 14.0 for statistical analysis. We ran frequencies to check for any inconsistencies in data entry. If any the inconsistencies were found, we verified them with the completed questionnaires which had a unique code. We calculated rates of *'ever smoker' *and *'current smoker' *among male and female students in each country according to our defined criteria. We presented the responses to the questions about students' practices regarding smoking among their patients seen during clinical rotations/clerkships as percentages for *'often' *and *'always'*. For the questions about the importance of topics in tobacco education module, we presented the results as percentages for *'important' *and *'very important'*. For questions about students' perceptions towards teaching about tobacco smoking we presented responses for *'agree' *and *'strongly agree'*. We also cross-tabulated these responses according to smoking status (*ever smoker *versus *never smoker*), gender and country. We used chi square test for statistical significance for observed differences between categorical variables. A p-value of less than 0.05 was considered as significant.

## Results

### Response rates and demographic characteristics

Overall response rate was 81.6%. The response rates in each country/site varied from 76% (Nepal) % to 83.2% (India). Median age of the students was 22 years (inter-quartile range 21, 23 years). Table 1 shows demographic information of the participants. Overall, the proportion of male and female students was 50.7% and 48.2% respectively. In all these sites the proportion of male and female students was nearly 50% except Pakistan (65% were females) and Nepal (67% were males). Distribution of students according to religion varied across the countries. Majority of the students were Muslims in Pakistan (99.4%), and Bangladesh (80.1%) while in Nepal the majority (86.2%) were Hindus. In India, students were Hindus (46.1%), Muslims (40.8), and Christians (10.6) while in Malaysia students were Hindus (29.5%) Muslims (26.5%), Christian (15.5%) and of other religions (24.0%).

**Table 1:** Response rates and demographic profile of the participants according to country (Number and percentage)

	Malaysia N = 200	India N = 208	Pakistan N = 161	Nepal N = 152	Bangladesh N = 201	Overall N = 922
Response rates	83% (200/240)	83.2% (208/250)	80.5% (161/200)	76% (152/200)	83% (201/240)	81.6% (922/1130)

Median age (years) inter quartile range	21 (20, 22)	24 (23, 25)	21 (20, 21)	22 (21, 23)	22 (21, 23)	22 (21,23)

**Gender**						

Male	95 (47.5)	96 (46.2)	56 (34.8)	102 (67.1)	118 (58.7)	467 (50.7)

Female	97 (48.5)	109 (52.4)	105 (65.2)	50 (32.9)	83 (41.3)	444 (48.2)

**Religion**						

Hindu	59 (29.5)	96 (46.1)	1 (0.6)	131 (86.2)	36 (17.9)	323 (35.0)

Muslim	53 (26.5)	85 (40.8)	160 (99.4)	1 (1.7)	161 (80.1)	460 (49.9)

Christian	31 (15.5)	22 (10.6)	0	2 (1.4)	0	55 (5.9)

Others	48 (24.0)	4 (2%)	0	18 (11.3)	3 (1.5)	73 (7.9)

**Residence**						

Hostel resident	69 (34.5)	45 (21.6)	77 (47.8)	64 (42.1)	178 (88.6)	274 (29.7)

Day scholar	93 (46.5)	158 (76.0)	84 (52.2)	86 (56.6)	19 (9.5)	598 (64.9)

**Selection criteria**						

Merit scholarship	63 (31.5)	48 (23.1)	11 (6.8)	36 (23.7)	26 (12.9)	184 (20.0)

Self-financed	108 (54.5)	147 (70.7)	150 (93.2)	113 (74.3)	169 (84.1)	676 (73.3)

Some of the percentages may not add up to 100% due to some missing entries in the demographic section of the questionnaire

### Self-reported smoking habits

Prevalence of smoking in all countries according to gender is presented in Table 2. Overall prevalence of *'ever smokers' *and *'current smokers' *was 31.7% and 13.1% respectively. Prevalence of 'ever smoker' was highest in Bangladesh (38.8%), followed by Malaysia (34.5%) and it was lowest in India (10.1%). Prevalence of smoking among males was higher than females in all countries which was statistically significant. Males students were more likely to be '*ever smokers' *(Unadjusted OR = 3.51; 95% CI 2.59 - 4.75) as well as '*current smokers' *(Unadjusted OR = 6.89; 95% CI 3.98 - 11.93). The difference in prevalence of smoking between countries was statistically significant for both '*ever smokers' *(p < 0.05) and '*current smokers' *(p < 0.01). Median age at initiation of smoking was 18 years which did not vary according to countries. Majority of current smokers smoked less than 10 cigarettes per day.

**Table 2:** Smoking habits among medical students according to gender and country

Country	Ever smoker			Current smoker		
	
	Male	Female	Total	Male	Female	Total
Malaysia(N = 200)	47 (49.5)	22 (22.7)	69 (34.5) *	18 (18.9)	4 (4.1)	22 (11.0)

India(N = 208)	19 (19.8)	2 (1.8)	21 (10.1) **	14 (14.6)	0	14 (6.7) **

Pakistan (N = 161)	24 (42.9)	22 (21.0)	46 (28.6) *	11 (19.6)	6 (5.7)	17 (10.6) **

Nepal (N = 152)	39 (38.2)	10 (20.4)	49 (32.2) *	23 (22.8)	4 (8.2)	27 (17.8) *

Bangladesh (N = 201)	60 (50.8)	18 (21.7)	78 (38.8) **	33 (28.8)	2 (2.4)	35(17.4) **

Overall (N = 922 )	206 (44.3)	81 (18.5)	292 (31.7) **	99 (22.5)	16 (3.9)	121 (13.1) **

*p < 0.05, **p < 0.001

Medical students' practices regarding smoking habits among their patients seen during most recent clinical rotations or clerkships are shown in Table 3. In all the countries, majority (> 80%) of the students asked the patients about their smoking habits, (duration and number smoked per day). About 40% of the students informed their patients about health effects of smoking. Only a third or less of the students either counseled or assessed willingness to quit smoking or assisted them in making a quit plan for their patients who were smokers. These practices varied significantly across the countries, but not according to smoking habits of the students. Female students were more likely to ask about smoking habits of patients and also inform them about health effects of smoking.

**Table 3:** Final year medical students' clinical practices towards tobacco smoking habits among their patients during their clinical rotations or clerkships according to their smoking status

		Ever smoker		Current smoker	
		
		Often (%)	Always (%)	Often (%)	Always (%)
1	Ask about history of smoking? ¶	67 (22.9)	179 (61.3)	19 (16.8%)	71 (62.85)

2	Ask about duration of smoking?	80 (27.4)	158 (54.1)*	25 (221.1)	62 (54.9)*

3	Ask about number of cigarettes/beedies smoked per day? ¶	61 (21.0)	165 (56.9)	19 (17.0)	69 (61.6)

4	Informed or advised patients about health effects of smoking? ¶	59 (20.3)	58 (20.0)	21 (18.8)	26 (23.2)

5	Counseled my patients who are smokers during clinical postings?	54 (18.6)	33 (11.4)	27 (23.9)	15 (13.3)

6	Assessed the willingness of the patient to quit smoking?	48 (16.5)	26 (8.9)	20 (17.7)	14 (12.4)

7	Assisted the patient to make a plan to quit smoking?	32 (11.0)	25 (8.6)	17 (15.0)	14 (12.4)

8	Informed patients about effects of passive smoking?	69 (23.7)	33 (11.3)**	27 (23.9)	20 (17.7)

*p < 0.05, **p < 0.001, ¶ these were statistically significant according to gender

Differences between the countries were significant for all the items p < 0.001

Table 4 presents medical students' perceptions regarding medical professionals' role in tobacco control and teaching about tobacco smoking in the medical curriculum. The results are shown as number and percentage of students who responded as either *'agree' *or '*strongly agree'*. Majority (> 80%) of the students had agreed about the following items: medical professionals have an important role in patients' smoking cessation, every patient should be asked about tobacco smoking, and all doctors should be competent about counseling and treatment for smoking cessation. Nearly 80% of the students agreed about the following items: medical professionals should be role models by being non-smokers to advice/counsel their patients, smoking among medical professionals is an obstacle for effective implementation of tobacco education and all medical schools should have facilities for smoking cessation. The perceptions of medical students about medical professionals' role in tobacco cessation were statistically significant according to smoking status and gender. Never smokers and female students were likely to respond as '*agree' *or '*strongly agree'*.

**Table 4:** "Mark your level of agreement with following statements about teaching on tobacco smoking in medical schools"? Number and percentage of students responding as 'agree' or 'strongly agree'.

		Agree	Strongly agree
1	Medical professionals play an important role on advising public/patients about smoking cessation? *	382 (41.4)	433 (47.0)

2	In clinical practice, tobacco smoking history should be routinely taken for every patient? *	319 (34.6)	533 (57.8)

3	All doctors should be competent to advise patients about, counseling & treatment of smoking cessation. ¶*	351 (38.1)	487 (52.8)

4	Medical professionals should be role models by being non-smokers to advice their patients smoking cessation. ¶*	231 (25.1)	490 (53.1)

5	All medical schools should have smoking cessation clinics with facilities for counseling, treatment & follow-up. ¶	362 (39.3)	375 (40.7)

6	Smoking amongst medical teachers and students is a main obstacle in effectively implementing tobacco education.	281 (30.5)	362 (39.3)

7	The current curriculum teaches adequately about health effects of active and passive smoking.	298 (32.3)	122 (13.2)

8	The current curriculum teaches about clinical guidelines, tobacco cessation methods and its contraindications.	258 (28.0)	94 (10.2)

9	All medical colleges should teach the students about cessation, treatment & counseling for smoking. ¶*	244 (26.5)	306 (33.2)

10	Current curriculum teaches about tobacco smoking but not systematic integrated with other disciplines departments.	326 (35.4)	92 (10)

11	All medical colleges should include tobacco education as a separate module in their curriculum.	295 (32.0)	166 (18.0)

¶these items were statistically significant according to ever smoker versus never smoker

* these items were statistically significant according to gender

For questions about tobacco education in their medical school curricula, only a third felt that they are being taught adequately about health effects of smoking, and tobacco cessation methods. Nearly half of the students felt that they should be trained about tobacco cessation methods including counseling; current training about tobacco smoking is not systematic and integrated with other disciplines. About half of the students agreed that medical school curricula should include a separate module about tobacco education. Three quarters of the students from Bangladesh agreed about teaching tobacco cessation, and counseling, in a separate module of the medical curriculum.

Students' perceived importance of contents (topics) in the tobacco education module is shown in table 5. Majority (> 80%) of the students indicated following topics as either *'important' *or '*very important'*: health effects of tobacco smoking (active and passive), symptoms of nicotine addiction, benefits of cessation, clinical guidelines for cessation, types of treatment and counseling techniques. About half of the students indicated epidemiology of smoking, clinical rotations and tobacco control policies and regulations as '*important' *or '*very important'*. About three fourth of the students indicated contents of cigarette smoke, indication and contraindications of cessation treatment, prevention of relapse as '*important' *or '*very important'*. There was very little variation across the countries, according to gender and smoking habits of the students.

**Table 5:** Rate the importance of following topics in tobacco education module in your curriculum

	Topic/contents of tobacco curriculum	Important	Very important
1	Epidemiology of tobacco smoking	322 (34.9)	195 (21.1)

2	Contents of cigarette smoke	356 (38.6)	323 (35.0)

3	Health effects of both active and passive smoking	230 (24.9)	595 (64.5)

4	Physical & psychological effects of nicotine addiction	255 (27.5)	557 (60.4)

5	Benefits from cessation of smoking	262 (28.4)	533 (57.8)

6	Clinical guidelines for smoking cessation	360 (39)	417 (45.2)

7	Types of cessation treatments available	384 (41.6)	378 (41.0)

8	Indications and contraindications for treatment	386 (41.9)	333 (36.1)

9	Counseling techniques to motivate patients quit smoking	331 (35.9)	416 (45.1)

10	Prevention of relapse and follow-up	387 (42.0)	331 (35.9)

11	Clinical postings in smoking cessation clinic	340 (36.9)	206 (22.3)

12	Tobacco control policies, regulations etc	310 (33.6)	306 (33.2)

## Discussion

Our exploratory survey from six medical schools in five South Asian countries has identified some gaps in medical students' practices about tobacco cessation and counseling. Self-reported use of cigarette smoking among medical students, students' perception about lack of adequate and systematic approach in training about tobacco smoking in their medical curriculum is a matter of concern. Interestingly, majority of the students indicated that contents of cigarette smoke, health effects of smoking and methods of smoking cessation and counseling as '*important' *in the tobacco module. Despite this only about half the students agreed that a separate tobacco module should be included in their curriculum.

Smoking rates among medical students in our survey were lower than those reported from previous surveys among medical students in Europe, North African and Middle Eastern countries [14-16]. Overall smoking rates in our survey were slightly higher than a previous survey in Asian countries [15]. The smoking rates among female students were lower in our study which is similar to the results reported from other surveys. In Asian countries, which are generally conservative societies, smoking is considered as unacceptable and thought to offend the social customs. However, as compared to previous surveys there is a slight increase in smoking rates among female students. This may be attributed to improvement in women's social status i.e. education, employment, urbanization and also marketing of lighter cigarettes meant for women by the tobacco industry [20]. We adapted questions about smoking habits from GHPSS while the studies cited above have adapted questions from WHO document. These studies varied from ours not only in classification of smokers and also the criteria used to define smokers.

Studies about smoking rates among practicing physicians are lacking. A survey from Kerala, India has reported that 10.8% of surveyed physicians were current smokers and 26% were *'ever smokers' *[21] which is higher than the smoking rates among students we surveyed from two medical schools in India. A survey from Lahore, Pakistan has also reported a higher smoking rates among physicians [22]. Though we did not find any literature about prevalence of smoking among physicians, we expect a similar pattern in other developing Asian countries. There is a need for leadership from the medical professionals by themselves being role models: "*doctor practice what you preach*" [23]. A smoking doctor is a poor role model for the patient. Regrettably, anecdotal evidence suggests that professional assistance or facilities to quit smoking habit are barely available in these medical schools for smoking doctors or medical students. The results of our survey support our argument in accordance with a worldwide medical school survey [24].

One of the important shortcomings among practices of medical students we surveyed was advising about health effects, counseling and smoking cessation for smokers. This may be due to lack of knowledge among medical students about smoking-related diseases and smoking cessation techniques [25]. The worldwide survey of medical schools has reported that medical curricula of medical schools in low and middle income countries are deficient in training about cessation techniques [24] unlike in USA [11] and other developed countries [13]. These findings are not surprising considering the results of a survey among physicians in Kerala, India which reported that only a third of physicians had received training about cessation methods [21]. In another study from Bahrain primary care physicians reported about insufficient training during their medical school [17]. Both GHPSS and global medical school survey have underscored the importance of training medical students about cessation techniques [9,24]. Even in our survey, students agreed that all doctors should be competent in counseling and cessation methods and about having tobacco cessation clinics in university teaching hospitals. The students' perceptions about medical professionals' role in tobacco smoking was optimistic and they agreed about deficiencies in their current curricula. However, this optimism of students does not convert into inclusion of a separate tobacco education module including training about cessation techniques. Possible reasons for such difference could be vastness of syllabus, current methods of teaching and contents of medical curricula in these medical schools. It is also possible that students in traditional lecture-based curricula consider these topics as an additional burden. We justify this from the results of our previous studies in two of the five medical schools where our survey was carried out. The students reported that *'vast syllabus' *and *'frequent examinations' *were important sources of stress [26,27].

Though a majority of the students in our survey indicated that health effects, nicotine addiction, and its treatment as important contents in the module on tobacco education, they did not favor an additional clinical posting in cessation clinics. One reason could be an additional burden on an overloaded student. Another possibility is non-availability or unawareness of such facilities in the teaching hospitals of these medical schools. However, we feel that medical educators should seriously consider about clinical postings during curriculum review for adoption of tobacco education module. Experts working on training of medical students or health professionals in tobacco control have suggested about several obstacles or barriers to effective implementation of tobacco curriculum. They have also suggested some solutions to overcome these obstacles [5]. Tobacco control experts, medical educators, administrative staff of medical schools, universities, accreditation bodies should work together in this direction. Further studies in each of these countries about current content of medical curriculum, mode of delivery, teaching learning outcomes and facilities for counseling and cessation treatment in the medical schools could be beneficial for revision of the current curriculum or introduction of a separate integrated tobacco education module. Such an initiative has been undertaken as a pilot project in India and Indonesia by the Project Quit Tobacco International [28]. Results of our survey support the need for such an initiative.

The results of our exploratory survey should be interpreted in the light of some limitations we had. Due to exploratory nature of the survey on a convenient sample of medical schools our results can only provide a snapshot about the medical schools we surveyed. Therefore, our results cannot be applied to other medical schools. However, our study is expected to set a benchmark for further studies about medical professionals' role and training medical students in tobacco control. As smoking behavior among students was self-reported there could have been reporting bias. Verification of self-reported smoking behavior with cotinine tests was not possible since our survey was not funded. Although participation in our survey was not compulsory, we obtained acceptable response rates. However, we cannot rule out some selection bias.

## Conclusions

Cigarette smoking was prevalent among medical students we surveyed. Counselling and cessation treatment is required for students who are smokers to quit their habit. Students were not practicing smoking cessation methods for their patients seen during clinical postings or clerkships. Though students have a positive perception towards medical professionals' role in tobacco control they were not encouraging about inclusion of a separate tobacco education module into their medical curriculum. However, they emphasised that health effects, counselling and treatment of nicotine addiction were important contents in the tobacco education module. Medical educators should consider about improving medical curricula to train tomorrow's doctors in prevention and cessation of smoking. Our results should be supported by larger surveys in more medical schools in each country.

## Competing interests

The authors declare that they have no competing interests.

## Authors' contributions

CTS: Conceptualized the research, wrote the first draft of questionnaire, and manuscript for publication; SS: Conceptualized the research, wrote the first draft of questionnaire, and manuscript for publication; RGM: Assisted in data collection, development and pretesting of questionnaire, commented on draft versions of the manuscript; HNHK: Assisted in data collection, development and pretesting of questionnaire, commented on draft versions of the manuscript; MR: Assisted in data collection, data entry, commented on draft versions of the manuscript; MRI: Assisted in data collection, development and pretesting of questionnaire, commented on draft versions of the manuscript; XVP: Assisted in development and pretesting of questionnaire, commented on draft versions of the manuscript; MS: Assisted in data collection and data entry, development and pretesting of questionnaire, commented on draft versions of the manuscript; BS: Assisted in data collection, data entry and analysis, commented on draft versions of the manuscript; US: Assisted in data collection, commented on draft versions of the manuscript; VRV: Assisted in data collection, commented on draft versions of the manuscript;

All authors read and approved the final manuscript to be submitted for publication.
